# Effects of an Immunosuppressive Treatment in the GRMD Dog Model of Duchenne Muscular Dystrophy

**DOI:** 10.1371/journal.pone.0048478

**Published:** 2012-11-21

**Authors:** Inès Barthélémy, Ane Uriarte, Carole Drougard, Yves Unterfinger, Jean-Laurent Thibaud, Stéphane Blot

**Affiliations:** Université Paris-Est, Ecole Nationale Vétérinaire d'Alfort, UPR de Neurobiologie, Maisons-Alfort, France; Instituto Butantan, Brazil

## Abstract

The GRMD (Golden retriever muscular dystrophy) dog has been widely used in pre-clinical trials targeting DMD (Duchenne muscular dystrophy), using in many cases a concurrent immune-suppressive treatment. The aim of this study is to assess if such a treatment could have an effect on the disease course of these animals. Seven GRMD dogs were treated with an association of cyclosporine A (immunosuppressive dosage) and prednisolone (2 mg/kg/d) during 7 months, from 2 to 9 months of age. A multi-parametric evaluation was performed during this period which allowed us to demonstrate that this treatment had several significant effects on the disease progression. The gait quality as assessed by 3D-accelerometry was dramatically improved. This was consistent with the evolution of other parameters towards a significant improvement, such as the clinical motor score, the post-tetanic relaxation and the serum CK levels. In contrast the isometric force measurement as well as the histological evaluation argued in favor of a more severe disease progression. In view of the disease modifying effects which have been observed in this study it should be concluded that immunosuppressive treatments should be used with caution when carrying out pre-clinical studies in this canine model of DMD. They also highlight the importance of using a large range of multi-parametric evaluation tools to reliably draw any conclusion from trials involving dystrophin-deficient dogs, which reproduce the complexity of the human disease.

## Introduction

Duchenne muscular dystrophy (DMD) is a recessive X-linked devastating muscle disease due to dystrophin-deficiency, affecting 1 newborn male in 3500, and for which to date no curative treatment exists. Affected boys exhibit progressive gait impairment, leading to the permanent use of a wheelchair usually by the beginning of their second decade, and the majority die in their third decade from respiratory or cardiac insufficiency [1].

The dystrophin-deficient dogs have been considered to be the most relevant model of the human disease and have consequently been used for pre-clinical trials targeting DMD. In addition to being a large animal model with a size approaching that of the young DMD boys, the dogs affected with dystrophin-deficiency are real canine counterparts of DMD patients, reproducing the multisystemic involvement and the severity of the human condition at the molecular, histological and functional levels [2], [3]. Thus many studies aiming to assess the efficacy of gene, cell or pharmacological strategies have been conducted in dog models of DMD [4]–[7].

During these studies, several of the investigators have been obliged to use immunomodulator treatments to avoid the immune response against the viral vector, donor cells and/or the product of a transgene [5], [7]–[12]. In this context, the drugs used were cyclosporine alone, or associated with mycophenolate mofetil, anti-thymocyte globulin or corticosteroids.

Numerous studies have however focused on the effect of immunomodulation in patients with DMD, and have, in a vast majority, demonstrated that similar pharmacological treatments do in fact modify the clinical evolution of the young patients. Corticosteroids, used at anti-inflammatory dosages, are known to improve the phenotype or at least to slow the disease evolution [13]–[17]. The effect of cyclosporine A at low dosages remains controversial, since it has been reported as beneficial by some studies [18], [19], and as non-existent by others [20].

Consequently a recent study conducted in GRMD (Golden retriever muscular dystrophy) dogs where cyclosporine and corticosteroids were used in conjunction with cell therapy [5] underwent severe criticism arguing that such an immunosuppressive treatment could alter the disease course progression and the effect of the tested therapeutic strategy [21].

Therefore it is essential to investigate the effects of these drugs administered alone at immunosuppressive levels on the pre-clinical model of DMD to see if there is any effect on the clinical outcome in these dogs. Despite the fundamental nature of this question, only one study has focused on the effects of prednisone in the GRMD dog, one of the most commonly used dystrophin-deficient dogs [22]. This study demonstrated that this treatment does indeed impact histological lesions and the muscle force.

In the present era of systemic therapeutic pre-clinical trials, it seems important to know what will be the exact influence of a concurrent immunosuppressive treatment on the global evolution of dystrophin-deficient dogs, from the histological to the systemic functional level. In order to better understand and interpret past and future results arising from pre-clinical trials, we decided to investigate the specific effect of the association of cyclosporine A and a corticosteroid, prednisolone, in the GRMD dog model. Here we present the results of a multi-parametric pharmacological trial, which aimed to provide reference data, allowing the reliable evaluation of the effect of therapeutic strategies in the trials necessitating concurrent immune suppression.

## Materials and Methods

### Ethics statement

All procedures were carried out in accordance with the *Guide of the care and the use of laboratory animals*, and approved by the common ethical committee of the ANSES, ENVA and UPEC, under the approval number 13/09/2011-7.

### Animals

Seven GRMD dogs were included in the study (Table 1). They were genotyped as previously described [23] before their inclusion.

**Table 1:** Summary of the dogs included in the study.

Name	Symbol (graphs)	Bioche-mical study	Clinical scoring	Biopsy 6 months	Biopsy 9 months	Force 4 months	Force 6 months	Force 9 months	3D-accele-rometry	Survival
**Clappy**	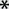	X	X	X		X				Death 6 months
**Clim**	**X**	X	X	X		X				Death 6 months
**Celsius**	**+**	X	X		X	X	X	X		Alive at treatment stop
**Cushing**	⧫	X	X	X	X	X	X	X	X	Alive at treatment stop
**Cvite**	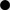	X	X	X	X	X	X	X	X	Death 9 months
**Doc**	▪	X	X	X	X	X	X	X	X	Death 9 months
**Doodle**	▴	X	X	X	X	X	X	X	X	Alive at treatment stop

**Figure 1:**
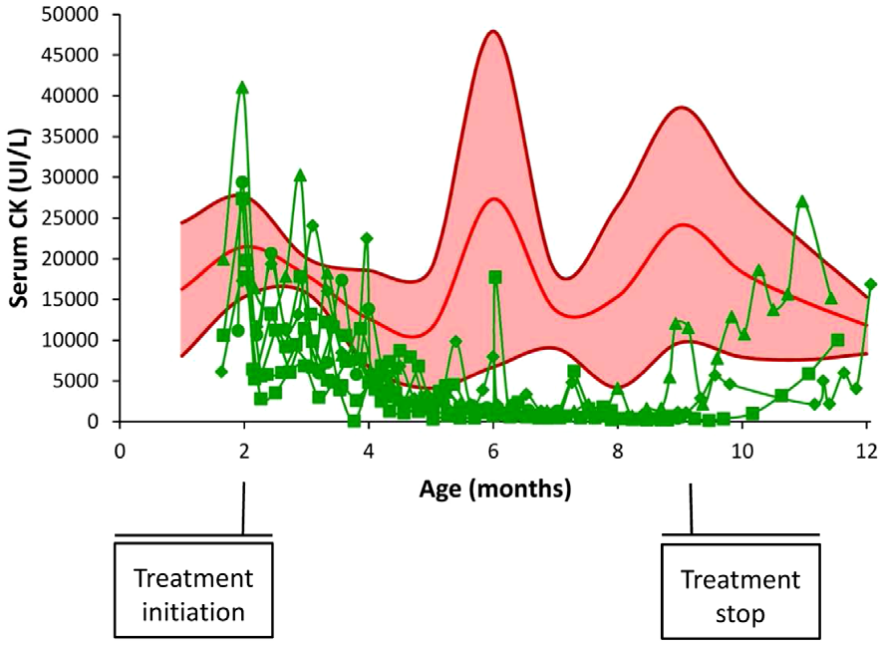
Evolution of the serum CK activity in the treated dogs. This graph represents the evolution of the serum CK in the treated dogs (green curves) in function of the age, before, during and after treatment in comparison with the mean value (red curve), ± SD (pink area) of 14 untreated GRMD dogs. The drop in the CK values during treatment and the re-increase after the treatment stop are remarkable.

**Figure 2:**
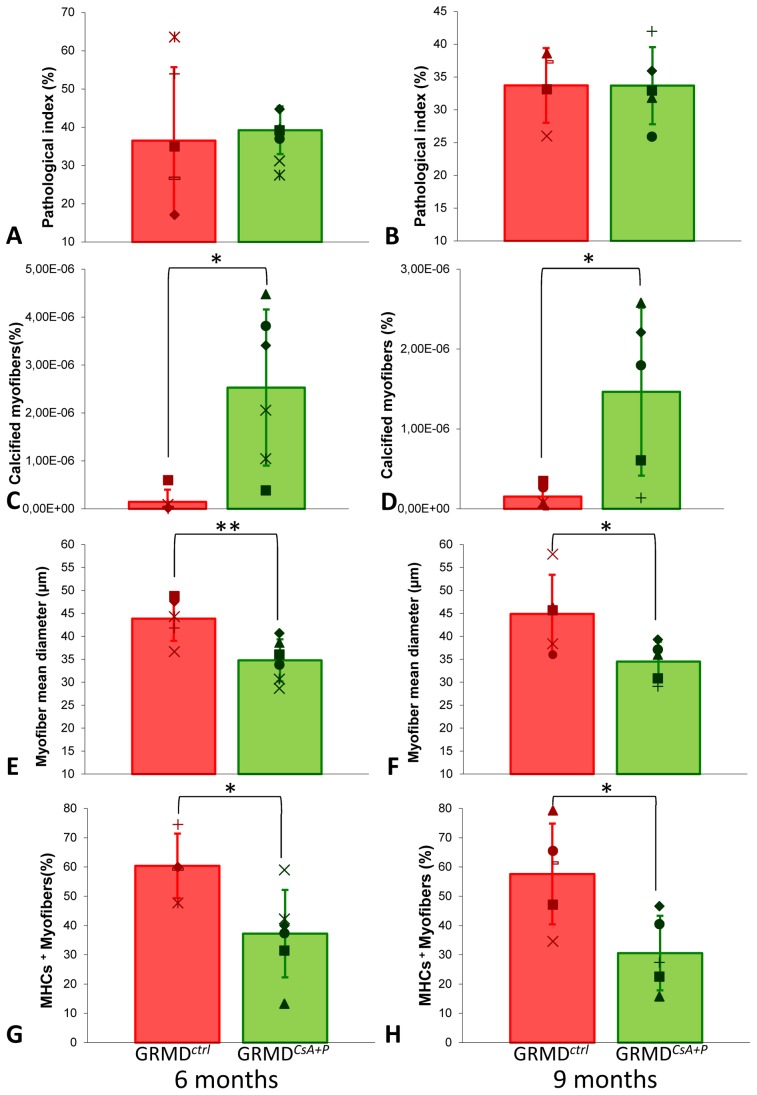
Main histological findings. This panel of graphs provides the main histological results arising from the quantitative analysis of the muscle biopsies. The mean ± SD of the obtained values are represented by histograms and error-bars, and because of the low number of biopsies analyzed, individual values are superimposed to the histograms. The results obtained in GRMD^ctrl^ dogs are in red on the left of the graphs, and the results obtained in GRMD^CsA+P^ are in green, on the right of the graphs. As biopsies were sampled at two points during the treatment, the values obtained at 6 months are represented in the left-hand graphs and the values at 9 months by the right-hand graphs. The pathological index was not significantly modified in the treated dogs, either at 6 months (A), or at 9 months (B). The amount of calcified myofibers was increased in treated dogs biopsies, at 6 (C) and 9 (D) months. The mean myofiber diameter was lower in treated dogs at 6 (E) and 9 (F) months. Finally, the biopsies originating from treated dogs contained significantly less slow-type fibers at 6 (G) and 9 (H) months. * means that p<0.05 ; ** means that p<0.01.

**Figure 3:**
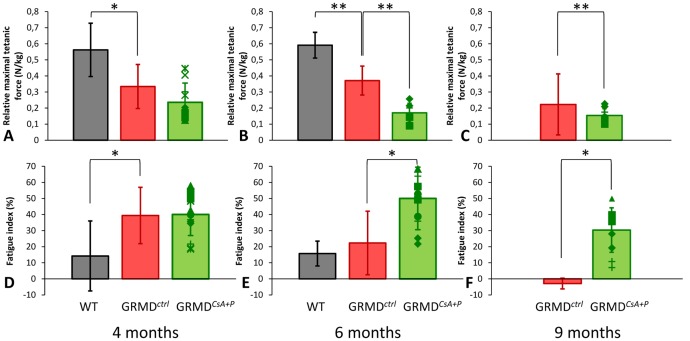
Main results of the in vivo force measurement. The results of relative maximal tetanic force (top graphs: A to C) and of the fatigue index (bottom graphs: D to F) are graphically represented at 4 months (on the left: A and D), 6 months (in the middle: B and E), and 9 months (on the right: C and F). The results obtained in the treated dogs (green) are compared with the ones obtained in the untreated GRMD dogs (red) and the healthy dogs (gray). The height of the histograms represent the mean value in the considered population, and the error-bar the SD. Right- and left-leg GRMD^CsA+P^ values are represented by superimposed points. The treated dogs had a significantly lower tetanic force at 6 (B) and 9 months (C), and an increased fatigue at 6 (E) and 9 months (F). Significant differences are represented by * if p<0.05 and by ** if p<0.01.

**Figure 4:**
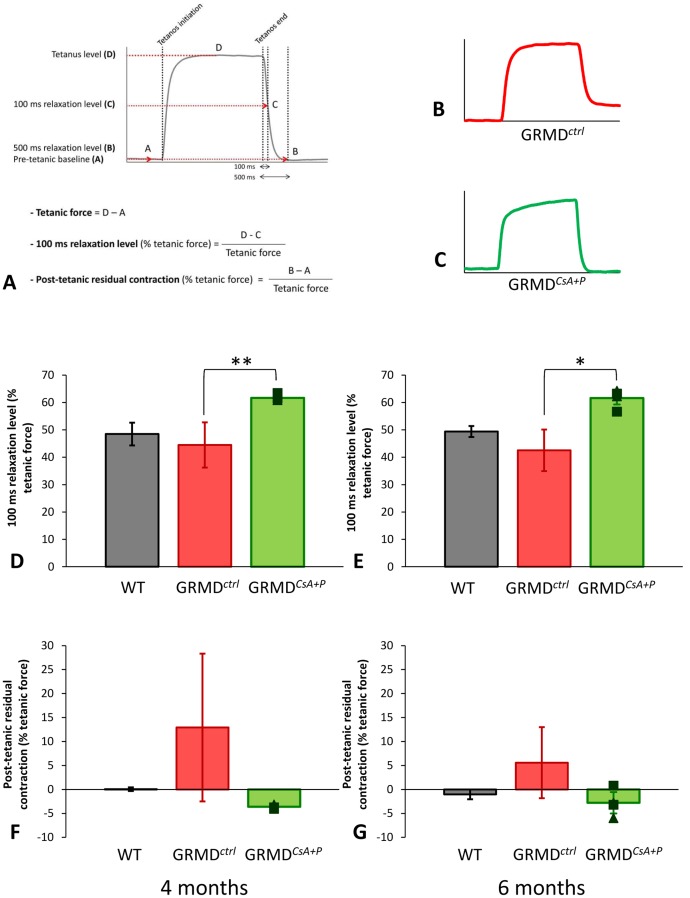
Post-tetanic relaxation patterns. The calculation method of the relaxation variables is schematized in the figure A. Figures B and C respectively represent the typical aspect of a tetanus in an untreated GRMD dog and in a treated GRMD dog. It is visible that a residual contraction is present in the case of the untreated dog (B), while the post-tetanic relaxation appears complete in the case of the treated dog (C). 4 month- (D and F) and 6 month- (E and G) relaxation index values obtained in the two treated dogs evaluated are represented in green, and compared with untreated GRMD (in red), and healthy dogs (in gray). The histogram height represents the mean value, ± SD (error-bar). The graphs on the top of the figure (D and E) represent the values of the 100 ms relaxation level, in percentage of the tetanic force value, whereas the graphs on the bottom represent the values of the post-tetanic residual contraction. The 100 ms relaxation level was significantly increased at 4 (D) and 6 months (E) in the treated dogs. The mean post-tetanic residual contraction was increased in untreated GRMD dogs, and decreased around healthy levels in treated GRMD dogs; however, these differences were not significant. Significant differences are represented by * if p<0.05 and by ** if p<0.01.

**Figure 5:**
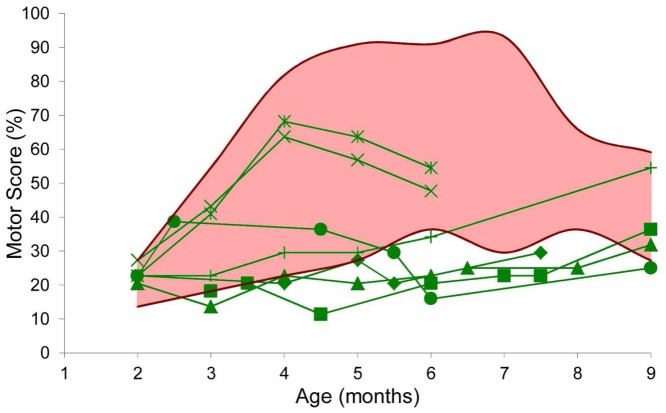
Evolution of the motor score during the treatment. This graph represents the longitudinal evolution of the motor score of the treated dogs (green, individual symbols provided in table 1), in comparison with the range (pink area) of values (dark red curves = min and max) obtained in a population of 24 untreated GRMD dogs, during the treatment period (2 to 9 months). Two features are uncommon and therefore noticeable: firstly the break in the curve of “Clim” [×] and “Clappy” [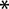], and secondly the unusually low values of the motor scores obtained in the 5 other treated dogs. The motor score were significantly improved in the treated dogs between 5 and 9 months.

**Figure 6:**
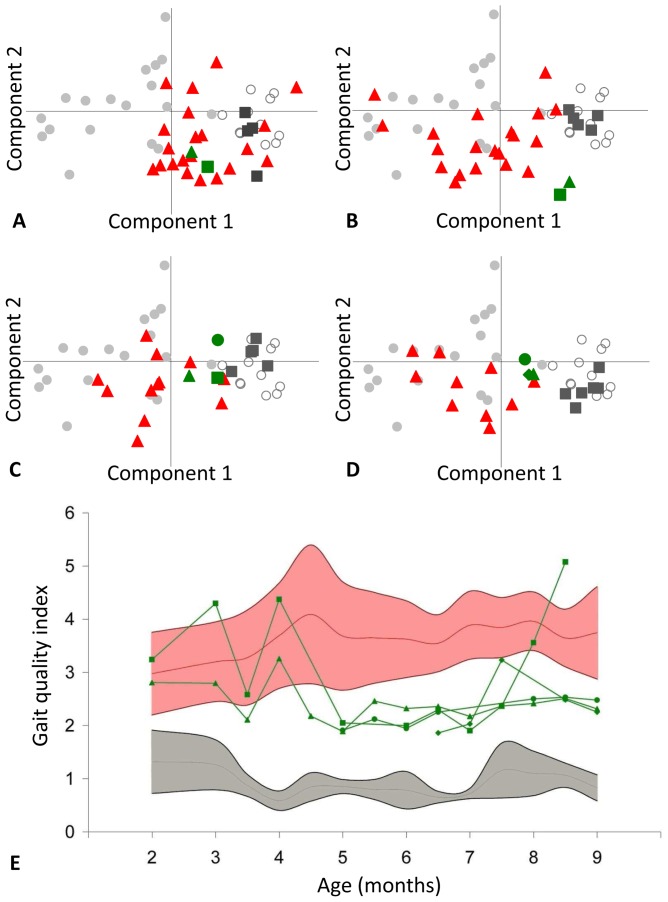
Gait evaluation during the treatment: global analysis by the study of the dog's course on the reference PCA plane quantified by the gait quality index. Position of the treated dogs (green own symbols) on the reference PCA plane defined by 18 adult GRMD dogs (grey circles) and 11 adult healthy dogs (empty circles), at 2 (A), 4 (B), 6 (C) and 9 (D) months. The age-matched untreated GRMD dogs are represented by red triangles and the age-matched healthy dogs by dark grey squares. The PCA was performed using 7 variables (stride frequency, stride length, total power, 3 relative axial powers, regularity), and the 2D-representation of the PCA induces a very low information loss, because it represents 90.58% of the variance (component 1: 68.17%, component 2: 22.41%). It is remarkable that the treated GRMD dogs, initially matched in position with untreated GRMD dogs, progressively move towards an intermediary position between GRMD and healthy dogs. This is quantitatively supported by the evolution of the gait quality index (graph E). The individual evolutions in treated dogs (green curves) are compared to the mean (red curve) ± SD (pink area) of untreated GRMD dogs and the mean (grey line) ± SD (grey area) of healthy dogs, in function of the age. The gait quality index is, from the age of 5 months in treated dogs, within intermediary values between untreated GRMD and healthy dogs. The position of the treated dogs is significantly nearer from the healthy center of gravity between 5 and 9 months.

**Figure 7:**
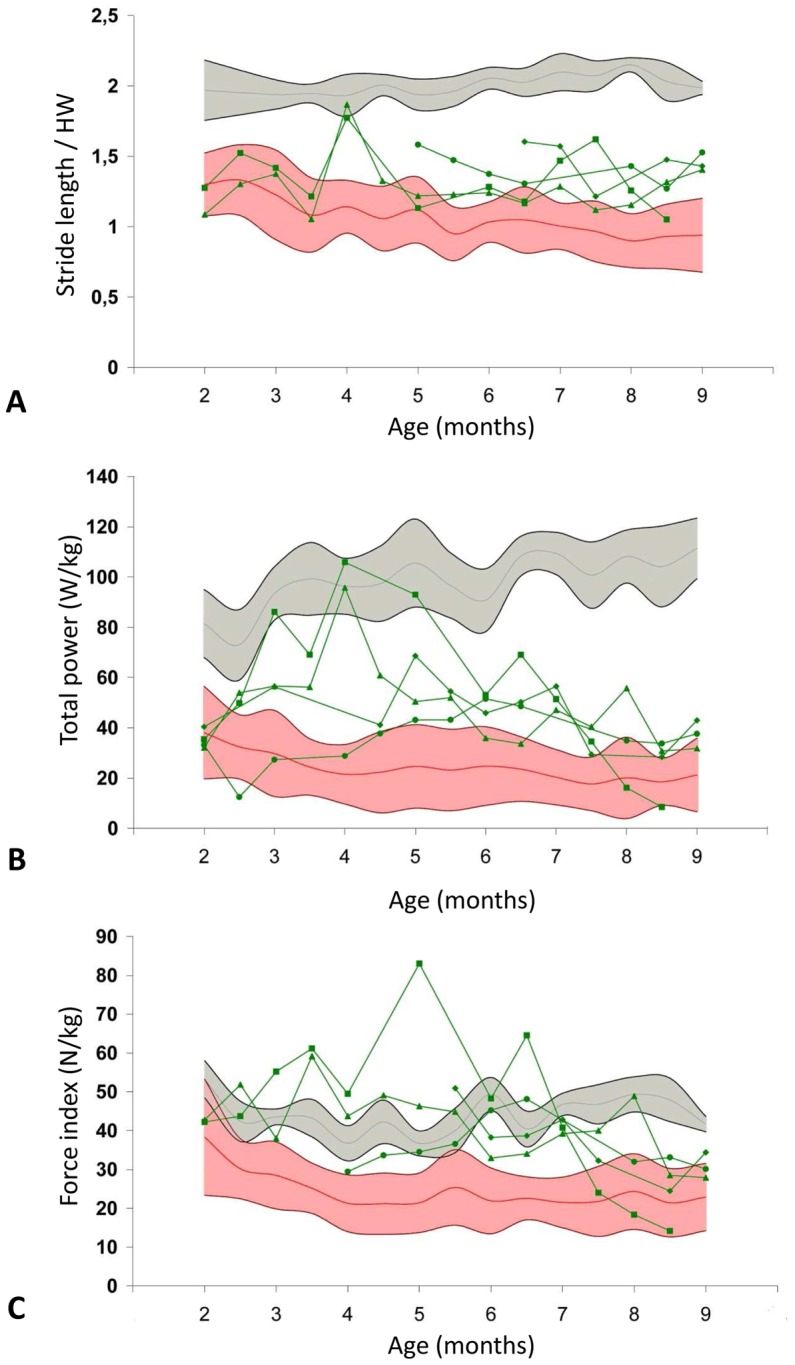
Evolution of three accelerometric variables during the treatment. The three graphs represent the evolution of the stride length normalized by the height at withers (A), the total power (B) and the force index (C), during the treatment. The treated dogs are represented by individual green curves, in comparison with the mean values (red curve) ± SD (pink area) obtained in untreated GRMD dogs and the mean values (grey curve) ± SD (grey area) obtained in healthy dogs. The improvement of the three variables during the treatment can be noticed on the graphs. These three variables become significantly improved in comparison with untreated GRMD dogs at many points between 3 and 9 months.

The table provides the main information about the different procedures carried out on each of the seven treated dogs. In the second column the individual symbols that will be used in the graphic representations (Fig. 1 to 7) are provided. A “X” present in a field indicates that the considered test was performed. The last column provides information on survival.

### Treatment

At 2 months of age, the immunosuppressant treatment was initiated. It was based on an association of high-dose prednisolone (2 mg/kg/d), and cyclosporine A (initial dose of 20 mg/kg/d). The treatment was administered orally, every 12 hours. The cyclosporinemia was controlled once to twice weekly and the cyclosporine A dosage was subsequently adjusted to maintain the trough cyclosporinemia level between 300 and 400 ng/ml, to ensure a suitable immunosuppression [24]. The treatment was continuous, until the dogs were 9 months of age.

### Clinical and biochemical follow-up

The dogs underwent a complete clinical examination daily. A weekly blood sampling for biochemistry was also performed, including a serum CK (creatine kinase) activity measurement. Fourteen untreated GRMD dogs were used as controls.

### Histological evaluation

#### Muscle biopsies

Biopsies were taken from right and left tibialis cranialis muscles of each dog at 6 and 9 months of age. Biopsies from 5 untreated 6 month-old and 9 month-old GRMD dogs were also taken and used as controls.

The dogs were anaesthetized using an intravenous induction of propofol (6.5 mg/kg), and was maintained with isoflurane in 100% O_2_. Analgesia was ensured by an intravenous injection of morphine (0.1 mg/kg). A continuous monitoring of ECG, SpO2, ETCO2, rectal temperature was set up, and an automatic ventilator was used to maintain normocapnia. Surgical biopsies were taken and immediately vertically mounted on a piece of cork, using tragacanth gum, and snap-frozen in isopentane cooled in liquid nitrogen. All samples were stored at −80°C prior to sectioning.

#### Histological analysis

The biopsies were sectioned at −28°C using a cryostat. Sections of 10 µm were used for morphological stainings, and sections of 7 µm for immunohistological experiments.

#### Histopathological evaluation using H&E staining

The dried sections were stained 10 minutes in Hematein and 5 minutes in 1% Eosin, dehydrated in four consecutive baths of ethanol, one bath of xylene and mounted in Canada balsam.

One entire section per biopsy was photographed using an AxioObserver Z1 linked to its ICC1 camera (Zeiss ®), and the MosaiX application of the software AxioVision (Zeiss ®). The image was then analysed using the software Visilog 6.4 (Noesis ®). A VBA (Visual Basic) program was created by the company Noesis, in order to quantify the dystrophic lesions, following the principle of a previously described method [25]. A grid of 10 000 µm^2^ squares was superimposed onto the image of the entire section. At each intercept of the grid (i.e. every 100 µm) the histological aspect of the underlying tissue was manually captioned using predefined annotations. The percentage of each type of histological event was calculated, as well as a pathological index, defined as the percentage of events not corresponding to normal shape fibers.

#### Evaluation of fibrosis using a picrosirius-fast green staining

The dried sections were fixed 30 minutes in 4% formaldehyde, rinsed and stained four minutes in a 0.1% Sirius red and 0.1% fast green saturated picric acid solution. They were rinsed in acidified water, dehydrated and mounted in Canada balsam. The Sirius red stained the collagen in red, and the fast green stained the cytoplasm in green. As for the H&E staining, the entire picrosirius-fast green stained sections were photographed and analysed using the software Visilog 6.4. A VBA program created by the company Noesis allowed an automatic measurement of red and green surfaces. The extent of fibrosis was calculated by dividing the red surface by the whole area of the section.

#### Evaluation of cytoplasmic calcium overload using Alizarin red S staining

The dried sections were stained for 5 minutes in a 2% alizarin red S solution at pH 5.4. They were rinsed and dehydrated in acetone and acetone-xylene volume/volume, and mounted in Canada balsam. The fibers with a normal calcium content appeared pale pink, whereas the calcium overloaded fibers appeared deep pink, to bright red for the more strongly overloaded. An entire section from each biopsy was photographed and the image was analysed using the software Photoshop CS3. A manual counting of the moderately overloaded (deep pink cytoplasm), and of the severely overloaded (red cytoplasm) fibers was carried out using the count tool. These two numbers, as well as their sum, were normalized to the number of fibers on the section. This number was estimated by counting the number of fibers on five randomly-selected fields of a known area representing a mean of 6% of the total section area, and by extrapolating this number to the whole section area.

#### Evaluation of the fiber diameter on ATP-ase stained sections

The dried sections were pre-incubated 10 minutes at pH 9.4, and 30 minutes in the presence of ATP at 37°C, and at pH 9.4. The ATPase activity was finally revealed by ammonium sulphide, after an immersion in a cobalt chloride solution.

Six different regions of the sections were photographed using a 10× objective. The images were analysed using the software Visilog, as well as a VBA program designed by the company Noesis, able to detect the outline of the fibers, and to calculate their equivalent diameter. The mean fiber diameter was used for the analysis, and the coefficient of variation was used to quantify the diameter inequality. At least 400 myofibers were counted on each section.

#### Evaluation of the fiber types using MHC immunostaining

Seven-micrometers sections from each biopsy were dried, rehydrated and fixed in cold acetone-methanol. After having blocked the endogenous peroxidase activity, the primary antibody (a mouse anti-human slow Myosin heavy chain (NCL-MHCs, Novocastra ®) was added onto the sections. Following washing in several baths of PBS a secondary HRP antibody was then added on the sections, positive binding was revealed with DAB.

Five randomly selected zones of the immunostained section were used for the analysis. The amount of MHCs^+^ myofibers were manually counted, and expressed as a percentage of the total number of myofibers counted in the sampled fields. At least 300 myofibers were counted on each section.

#### Evaluation of the inflammatory infiltration using CD4, CD8, and CD11b immunostainings

Seven-micrometer sections from each biopsy were dried, rehydrated and fixed in cold acetone-methanol. After having blocked the endogenous peroxidase activity, the primary antibody (either a rat anti-canine CD4, Serotec ®, 1/50, or a rat anti-canine CD8, Serotec ®, 1/50, or a mouse anti-canine CD11b, Serotec ®, 1/50) was added onto the sections. Following washing in several baths of PBS a secondary HRP antibody was then added on the sections, positive binding was revealed with DAB.

CD4^+^, CD8^+^ or CD11b^+^ cells were counted on each entire section. The amount of inflammatory cells was normalised to the area of the section.

A Student t-test between treated and untreated dogs was used at 6 and at 9 months, to investigate the effect of the treatment on the histological lesions. The level of significance was set at p≤0.05.

### In vivo force measurement

The in vivo force measurement was performed at the age of 4, 6 and 9 months, as previously described [5]. Briefly the dogs were anaesthetized, following the same protocol as for the muscle biopsies. They were positioned in dorsal recumbency and both their hindlimbs were successively positioned into a dedicated device already used to assess muscle force after therapeutic procedures [5], [26]. The device is composed of two force transducers (Grass transducers FT10) linked to a vertical plate supporting the anatomical segment encompassing the tarsus, metatarsus and digits. The tarsus was flexed to 90°, so that the leg rested horizontally, the femor was maintained in a vertical position by gently maintaining a 90° flexion of the knee. A percutaneous stimulation of the fibular nerve was performed, and the electromyographic activity was controlled in order to ensure supramaximal stimulation, which was reached by progressively increasing stimulation intensity using 2 second- tetanic stimulations at 50 Hz. Once the supramaximal stimulation intensity was fixed, 6 1-minute-spaced tetanic stimulations were applied, leading to the contraction of the cranial compartment of the leg inducing a tarsal flexion and a digital extension. The signal produced by the transducers was amplified (Grass CP122 amplifiers), and digitally converted and recorded (iWorx 214 and Labscribe software). The maximal tetanic isometric force was obtained by averaging the values of the six tetanic contractions. For the analysis, and to compare the animals, this absolute force value was normalized to the body weight of the dog, leading to a relative force index (N/kg).

The values obtained in the 4, 6 and 9 month-old treated dogs were compared to those measured respectively in 8, 5 and 2 age-matched untreated GRMD dogs.

After the acquisition of the 6 tetanic contractions, 40 3-second-spaced tetanic contractions were induced in order to evaluate fatigue. The fatigue index (%) was calculated by normalizing the absolute force of the last tetanus by the absolute force of the first tetanus. The results were compared with those obtained in the same population as for the relative force.

Finally, the post-tetanic relaxation was studied in two of the treated dogs. The speed and level of immediate post-tetanic relaxation were evaluated using two indexes: the 100 ms relaxation level, expressed as a percentage of the tetanic absolute force, and the post-tetanic residual contraction, calculated as the difference between the baselines before and 500 ms after the tetanic contraction, and expressed as a percentage of the tetanic absolute force. The values obtained on the two 4 and 6 month-old treated dogs were compared with those obtained respectively in 30, 11 and 9 age-matched untreated GRMD dogs.

As values from both legs were available for all the calculated variables, the side (left versus right) was used as a within effect in an ANOVA, the between effect being the group (treated versus untreated GRMD). The level of significance was set at p≤0.05.

### Clinical scoring

The treated dogs underwent a monthly clinical scoring using the grid which had been previously developed and validated in the laboratory [27], and this has been provided as Figure S1. The scores which we obtained were compared to the values obtained in 24 age-matched untreated GRMD dogs, using a t-test. The level of significance was set at p≤0.05.

### Evaluation of gait quality using 3D-accelerometry

The gait quality was quantified using a previously described method based on 3D-accelerometric recordings near to the centre of gravity during spontaneous locomotion [28]. Briefly the dogs were allowed to walk or run along a corridor at a self-selected gait and speed. During the test, 3 orthogonally-positioned accelerometers inserted in a small device (Locometrix ®) were maintained near to the xiphoid process using a thoracic belt, and recorded accelerometric curves (acquisition frequency 100 Hz).

A dedicated software (Equimetrix ®) was used to calculate the following variables, described as relevant in growing GRMD dogs: the speed, the stride frequency, the stride length, the regularity, the dorso-ventral, cranio-caudal and medio-lateral powers, and the total power. The speed and stride length were used after normalization by the height at withers, and the axial powers were expressed as relative powers after a normalization by the total power. A force index was also calculated by dividing the total power by the speed.

Finally, a global index, named gait quality index (GQI), was calculated as the Euclidean distance of each dog to the centre of gravity of age-matched healthy dogs, projected as supplementary individuals on a previously described PCA plane [29]. This PCA was built using 7 variables: the stride frequency, stride length, regularity, the three relative powers and the total power. Thus the GQI quantitatively reflects the deviation of the GRMD dogs from the normal situation regarding 7 accelerometric variables.

The tests were performed every two weeks, from the age of 2 months, to the age of 9 months. However, due to the fact that the accelerometry method to evaluate the gait of GRMD dogs was not available at the beginning of this study, only four of the seven treated dogs could be evaluated using this tool. Among these four dogs, due to the progressive evolution of the methodology we have only partial data (speed not measured) for two of them (Cushing and Cvite). The four treated dogs were compared to 24 untreated GRMD dogs (the same dogs as for the clinical score), evaluated using the same method. The statistical comparison was made using a t-test at each time point. The level of significance was set at p≤0.05.

## Results

### Study course and clinical follow-up

Among the seven dogs included, two (Clim and Clappy) died before the end of the treatment from acute pneumonia at the age of 6 months, the day before the planned force measurement. All of the measures were made on the five other dogs. However, two of them (Cvite and Doc) were in a bad shape at the end of the treatment, and Doc was unable to perform the last 3D-accelerometry test at 9 months. They both died during recovery from anaesthesia from pulmonary haemorrhage, most probably due to a coagulopathy induced by a hepatic insufficiency (cyclosporine dysmetabolism, low uraemia, elevated hepatic enzymes in the serum). These symptoms were just part of the numerous secondary effects of the treatment observed in the treated animals. Side effects included gingival overgrowth, extended viral-induced papillomas, ectopic calcifications, overweight, growth retardation, and opportunist bacterial infections.

Usual medical complications related to GRMD were also observed: hiatal hernia (4/7 dogs), dysphagia justifying the placement of a gastrostomy tube (1/7), aspiration pneumonias (3/7), severe joint luxations and/or deformities (3/7).

The table 1 summarizes the evaluation protocol carried out in each dog, as well as the main events that occurred during the treatment period.

The table S1 provides the main results of the multi-parametric evaluation of the treated dogs at 2, 4, 6 and 9 months of age.

### Decreased serum CK

The treated GRMD dogs (GRMD*^CsA+P^*) exhibited strongly increased serum CK activity values at the beginning of the treatment, in accordance with those observed in the GRMD control (GRMD*^ctrl^*) population. However, these values progressively decreased during the two first months of treatment, to reach unusually low values at 4 months, and remained low until the end of the treatment (figure 1). For example, at 6 months (4 months of treatment), the mean CK activity value in the GRMD*^CsA+P^* was 1445.8 UI/L (SD: 1267.1 UI/L), a dramatically low level in comparison to the mean age-matched GRMD*^ctrl^* value: 27326.7 UI/L (SD: 20605.5 UI/L) (p = 0.005).

Interestingly, the dogs who survived after the end of the treatment showed a subsequent increase of their CK values that reached GRMD*^ctrl^* levels within a few weeks (figure 1).

### Histological features

#### Unmodified histological lesions

No differences were observed in the GRMD*^CsA+P^* and GRMD*^ctrl^* biopsies, following the histopathological assessment of the H&E stained sections (figure 2 A and B), either in the amount of fibrosis, the variability in the fiber diameters, or the inflammatory infiltrate (p>0.05), whatever the age (6 or 9 months).

#### Increased number of calcified myofibers

The quantification of calcified myofibers using the Alizarin red S specific staining demonstrated a significant increase in the number of these lesions in GRMD*^CsA+P^* biopsies (figure S2), at 6 (p = 0.011) and 9 months (p = 0.024) (figure 2 C and D).

#### Decreased myofiber diameter

The mean myofiber diameter was found to be significantly smaller in GRMD*^CsA+P^* compared to GRMD*^ctrl^* biopsies at both 6 (p = 0.003) and 9 months of age (p = 0.042) (figure 2 E and F).

#### Decreased type I fibers proportion

The percentage of slow MHCs^+^ positive fibers was significantly decreased in biopsies of 6 (p = 0.030) and 9 month-old (p = 0.022) GRMD*^CsA+P^* dogs (figure 2 G and H, figure S3).

### In vivo force measurement

#### Decreased tetanic force

GRMD*^ctrl^* dogs already show a significantly decreased relative tetanic force compared with healthy dogs. However, the mean GRMD*^CsA+P^* relative tetanic force was even lower than in the GRMD*^ctrl^* at all three ages (4, 6 and 9 months). This difference was found to be significant at 6 (p = 0.001) and 9 months (p = 0.001) (figure 3 A, B and C).

#### Increased fatigue

The fatigue index significantly increased in GRMD*^CsA+P^* dogs compared with GRMD*^ctrl^* dogs, at 6 (p = 0.028) and 9 (p = 0.026) months (figure 3 D, E and F).

#### Improved post-tetanic relaxation

The post-tetanic relaxation speed, evaluated in this study by the 100 ms relaxation level, was slightly reduced in GRMD*^ctrl^* relative to healthy dogs, at the age of 4 and 6 months. This index was significantly increased in GRMD*^CsA+P^* dogs compared with GRMD*^ctrl^* at 4 (p = 0.001) and 6 (p = 0.016) months. This 100 ms relaxation level was even higher in GRMD*^CsA+P^* dogs than in healthy dogs (figure 4 D and E).

A common feature of post-tetanic relaxation in GRMD*^ctrl^* dogs is an uncomplete relaxation, resulting in a residual post-tetanic contraction, quantified in this study as a percentage of the tetanic force. This index was reduced to normal levels (near 0%) in the two tested GRMD*^CsA+P^* dogs, at 4 and 6 months (figure 4 F and G). However the difference was not significant (p = 0.117 and p = 0.170, respectively).

Briefly the post-tetanic relaxation was found to be quicker and more complete in GRMD*^CsA+P^* dogs.

### Improved motor score

The progression in the motor score of the GRMD*^CsA+P^* dogs during treatment appears unusual (figure 5). Two animals (Clim and Clappy) exhibited a rapid increase of their motor score during the two first months of treatment, followed by a decrease during the following two months, until their death. This suggests a motor improvement in severely affected and rapidly evolving dogs. Such a break in the motor score curve is unusual in GRMD*^ctrl^* dogs. The five other GRMD*^CsA+P^* dogs exhibited a tiny increase of their motor score during the 7-months treatment period. Between the age of 5 and 8 months, the GRMD*^CsA+P^* motor score values were below the minimal values obtained in the 24 GRMD*^ctrl^* dogs. For example, at the age of 6 months (4 months of treatment), the mean motor score value in the GRMD*^CsA+P^* was 32.6% (SD : 15.7%), i.e. approximately half of the GRMD*^ctrl^* value: 63.8% (SD: 20.0%) (p = 0.003).

The GRMD*^CsA+P^* motor scores were found to be significantly improved during the four last months of treatment (between the age of 5 and 9 months).

### Improved gait quality

A first approach to globally analyse the 3D-accelerometry data consists in examining the trajectory of the GRMD*^CsA+P^* dogs on the reference PCA (principal component analysis) plane described above, throughout the treatment period. The position of the individuals on this plane reflects their gait quality measured by seven different variables.

The GRMD*^CsA+P^* dogs were superimposed on the GRMD*^ctrl^* cloud at the beginning of the treatment (2 months), but progressively left the GRMD*^ctrl^* cloud to approach the healthy cloud, and they maintained this particular position between 6 and 9 months of age (figure 6 A to D). This trajectory has been quantified using the gait quality index (GQI). During the first weeks of treatment the GRMD*^CsA+P^* dogs could not be distinguished from the GRMD*^ctrl^* dogs regarding the GQI. However, from five months of age, the GRMD*^CsA+P^* dogs showed a decreased GQI, with values which were intermediate between the healthy and the GRMD*^ctrl^* populations (figure 6 E). For example, at 6 months of age, the mean GRMD*^CsA+P^* GQI was significantly decreased (p = 0.003) to 2.09 (SD: 0.20), a value which represents the approximate mid-distance between the mean GRMD*^ctrl^* position (3.62 SD 0.72) and the mean healthy position (0.78 SD 0.35). Similarly the decrease in motor score was found to be significant from the age of 5 to 9 months, except for of the dog “Doc” who showed a sudden gait deterioration at the 8.5 month point, probably linked to a severe general decline in health. These results clearly demonstrate that there is a global improvement in the gait quality of the GRMD*^CsA+P^* dogs, that should be better characterized by the detailed analysis of the gait variables.

All the variables which we investigated were significantly improved during the treatment period. However, some of them were more dramatically improved than others. One of the most striking gait modifications was the increased stride length, normalized to the height at the withers, in GRMD*^CsA+P^* dogs. This value which was strongly decreased at the beginning of treatment gradually improved for the GRMD*^CsA+P^* dogs who, progressively, became able to lengthen their strides to reach intermediate values between GRMD*^ctrl^* and healthy dogs (figure 7 A). The stride length of these treated dogs was thus significantly increased at 4 (p<0.001), 5.5 (p = 0.014), 6 (p = 0.009) and between 7 and 9 months (0.001<p<0.030). The stride frequency was not strongly influenced by the treatment, therefore the increase in stride length alone was responsible for the significant increase in speed observed, at 4 (p<0.001), and between 7 and 9 months (0.003<p<0.030).

This increase in speed may contribute in part to the improvement of the GRMD*^CsA+P^* total power. This very discriminant variable in GRMD*^ctrl^* dogs was decreased in GRMD*^CsA+P^* dogs before the treatment was initiated. However, during the treatment a progressive increase in the total power was observed in GRMD*^CsA+P^* dogs, and this variable was maintained at a level which was intermediate between GRMD*^ctrl^* and healthy dogs, almost until the end of the follow-up (figure 7 B). Moreover, this increase in total power is statistically significant between 3 and 7.5 months (0.0001<p<0.023).

Interestingly, with regard to the evolution of the force index, this increase in power cannot be entirely explained by an increase in speed. This index was calculated by normalizing the total power by the speed, in order to overcome the power variations linked to speed differences. The force index was significantly increased in GRMD*^CsA+P^* dogs between 3 and 7 months (0.0001<p<0.009), showing that the treatment makes the gait more powerful, independent of the gait speed (Figure 7 C).

## Discussion

Undoubtedly, this study has shown that the association of cyclosporine A and prednisolone as an immunosuppressive therapy does indeed have an effect on the GRMD disease course and at many levels.

The dominant effect is a significant improval in motor function. Indeed the motor scoring, as well as the gait analysis by 3D-accelerometry clearly agreed to characterize the treated dogs as mildly affected. The motor scores were much lower than any previously quoted values in GRMD dogs, and primary alterations in gait were at least partially corrected by the treatment. This probably reflects the fact that the double immunosuppressive therapy must have a positive effect on contractures, already described in GRMD dogs treated by prednisone alone [22]. Indeed, the motor score quantifies contractures in many items and the stride length, which quantifies the amplitude of movements, is also an indicator of contractures [28], [30].

These reduced contractures may be related to the improvement of relaxation, also documented in this study. In GRMD dogs the relaxation troubles are strongly correlated with the motor score (unpublished data) and interestingly, this study suggests that an improvement in relaxation could be associated with an improvement both in motor score and gait quality. This implies that the impairment in relaxation could play an important role in the pathogenesis of GRMD. It may be hypothesized that these features could be related to defects in ionic exchanges and homeostasis in the dystrophic myofiber [31], [32]. The immunosuppressive treatment could have contributed to improve these ionic abnormalities, and to decrease the necrotic activity, leading to the dramatic decrease in the serum CK in these dogs with a preserved mobility [33], [34]. Beyond this study, these results argue in favour of therapeutic strategies for DMD, targeting the ionic homeostasis.

In contrast to these beneficial effects, histological and force measurement features were not favorable.

From a morphological point of view, the muscle biopsies of treated dogs were characterized by numerous calcified myofibers. This observation was also reported in GRMD dogs under high-dose prednisone [22]. Calcified myofibers is a common dystrophic lesion in dogs [35], but abnormally prominent in the treated animals. The authors of the previous prednisone study hypothesized that the accumulation of these fibers was due to a decreased macrophagic activity [22]. The CD11b staining performed in the present study has shown that the total number of CD11b positive cells was unchanged in treated GRMD dogs, but it was remarkable to notice that the calcified myofibers were not found in macrophage-rich zones, conversely to what is usually seen in biopsies of the untreated GRMD dogs. This observation is in favour of the hypothesis formulated by Liu et al.

Another observation made on the muscle biopsies was that there was a switch of myofibers towards the fast type. This is most probably a consequence of the inhibition of calcineurin activity in muscle fibers [36], [37], and may explain the increased fatigue after repeated tetanus, the increased relaxation speed, and maybe even the decreased tetanic force. It is already known that dystrophin-deficient fast fibers are less resistant to exercise than slow fibers, maybe explaining the slow type predominance described in GRMD dogs and DMD patients [5], [38].

The decreased tetanic force, also reported in high-dose prednisone treated GRMD dogs [22], could also be a consequence of the smaller fiber diameter and atrophy which we have observed. This atrophy can be mediated by inhibition of the calcineurin pathway and also by corticosteroids [39]–[41]. It is interesting to note that inducing muscle fiber atrophy in the pre-clinical model of DMD does not alter the global motor phenotype, giving less weight to strategies targeting atrophy for DMD. This conclusion was also recently corroborated by a report on the phenotype of myostatin-null heterozygote GRMD dogs, showing that muscle hypertrophy is deleterious in this model of DMD (J.N. Kornegay, oral communication at 4th Annual Symposium of the ESVN Neurological Genetic Diseases Trier, 23–24 september 2011).

A final explanation for the decrease in force is a critical methodological point: the normalization of the tetanic force to the body weight. Conventionally the tetanic force is normalized to the body weight in GRMD dogs to avoid artefactual variations of force due to different sizes. However, GRMD dogs under immunosuppressive treatment were overweight, whereas most of untreated GRMD dogs were underweight [2]. This could have contributed to widen the gap of force between untreated and treated GRMD dogs. This problem, also raised by Liu et al. [22], could be solved in the future by normalizing the absolute force to the size of an anatomical element or the volume of the analysed muscle group.

Some other limitations of this study may be pointed out. First the fact that the dogs were treated with two different drugs, and that no dog was treated with cyclosporine only or prednisolone only, makes the interpretation of the results more difficult. Indeed the observed modifications cannot be attributed to one molecule or the other, even though the effect of high dose prednisone (2 mg/kg) has been documented in one study [22]. However the purpose of this study was not to evaluate the potential therapeutic effect of these drugs, but to monitor their associated effect at immunosuppressive levels, as they are used as a concurrent treatment in therapeutic pre-clinical trials.

Another limitation of this study is the heterogeneity of the functional evaluation schedule between dogs. Indeed if all the dogs underwent muscle biopsies and force measurement, the same does not hold true for accelerometric measurements for example. The seven GRMD dogs were included in the protocol within one year and a half, during which some new evaluation tools were developed, notably 3D-accelerometry. The second half of the dogs included in the study were thus evaluated using this method, and we chose to present the data, even if obtained on only a small number of dogs, because of their significance.

Despite these few methodological limitations, it is possible, in the light of our results, to give an answer to the criticism made against one of the studies in which a combined immune suppression using cyclosporine and corticosteroids was used in GRMD dogs [5], [21]. First, the unilaterally increased tetanic force in the injected leg, reported in this study, cannot be attributed to the combined immunosuppressive treatment which has been shown in the present study to decrease muscle force. However, it is not possible to associate the global motor improvement observed in the grafted dogs to a stem cell-associated benefit. Indeed, at the time when this study was performed, none of the tools to quantitatively evaluate GRMD locomotion were available [28], [30]. It is thus impossible retrospectively to assess if the observed improvement was even better than that induced by immune suppression only.

In addition, the immunosuppressed GRMD dog must be considered as a completely different model from the untreated GRMD dog, since so many functions are deeply modified. Notably in a context of a systemic therapeutic trial, it can be assumed that it may be difficult to clearly demonstrate a locomotor benefit with such a beneficial immunosuppressive treatment. In addition, these drugs can impact biodistribution, notably cell migration properties [42].

In view to translate these results from the GRMD dog to DMD patients, one can suppose that despite the known benefit provided by steroids at anti-inflammatory dosages in humans, the increase of their dosage to reach immunosuppression and their combination with another immunosuppressive drug would impede muscle force and pathology. More importantly, knowing these effects on the natural course of the disease will improve the relevance of the conclusions obtained in GRMD treated with cell or gene therapy under immunosuppression and therefore improve the translation of data.

In conclusion, the combination of cyclosporine A and prednisolone at immunosuppressive levels has a strong effect on the GRMD phenotype, leading to the exacerbation of calcification lesions and atrophy, to a decreased muscle force, and increased fatigue, but surprisingly to a global improvement of the disease progression at the systemic level, with decreased CK values and improved locomotion. If only the histological features and force had been evaluated, as is often the case in many trials, one would have concluded to a deleterious effect of the treatment. This underlines the interest of a multi-parametric and global evaluation of this canine model of DMD, which reproduces the complexity of the human disease. Therefore, the same would probably hold true for clinical trials on DMD patients.

A last conclusion that can be drawn from this study is that, despite the widely described GRMD clinical heterogeneity, which often brings this model bad press [3], [43], it is however possible to clearly demonstrate, using adapted tools, an effect of a treatment on a complex function such as locomotion, and on a reasonable number of individuals. One thing to keep in mind, when designing a pre-clinical trial involving GRMD dogs, is that the expected effect of the tested treatment must be significant, to be reliably detected it small cohorts. This last point makes even more sense since this precious model must be used to validate a clear gain of functionality, as it is a key actor required to translate treatments to DMD patients.

## Supporting Information

Table S1 **Main results of the multi-parametric evaluation of treated versus healthy and untreated GRMD dogs.** The mean (SD) of each evaluated parameter is provided in this table, at 4 time points of the study: from treatment initiation (2 months) to treatment end (9 months), with two intermediary points (4 and 6 months). The results are given for the treated dogs (GRMD^CsA+P^) and for the untreated GRMD dogs (GRMD^ctrl^), as well as for the healthy population when this last information is available.
(DOCX)

Figure S1 **Clinical motor scoring grid.** The clinical motor scoring grid encompasses 11 items, allowing the operator to evaluate the dog by observing postural abnormalities, contractures, ambulation, or by performing simple tests as the hopping test or the ability to stand up or cross an obstacle. Each item can be scored from 0 (normal situation) to 2 (the worst situation), giving a score on 22 points, the score 0/22 being the one a healthy dog should obtain, and 22/22 describing the worst motor clinical situation a GRMD could be in. Abbreviations: IDS: inter-digital space; from lat. rec.: from lateral recumbency.
(TIF)

Figure S2 **Illustration of the high prevalence of calcified myofibers in treated dogs.** A: Macroscopically visible calcified myofibers in a GRMD^CsA+P^ tibialis cranialis muscle. B: Alizarin red S (ARS) staining of a GRMD^CsA+P^ tibialis cranialis muscle biopsy at 6 months, picture of a whole section. The amount of ARS positive fibers is remarkable.
(TIF)

Figure S3 **Illustration of the MHCs+ positive fibers rarefaction treated dogs.** MHCs immunostaining of a whole GRMD^ctrl^ tibialis cranialis biopsy section (A) in comparison with the same immunostaining performed on a whole GRMD^CsA+P^ tibialis cranialis biopsy section at 9 months (B). The loss of MHCs^+^ fibers predominance in treated dogs is evident.
(TIF)
