# Supplementary material for: Effects of an Immunosuppressive Treatment in the GRMD Dog Model of Duchenne Muscular Dystrophy
Source: PLoS One. 2012 Nov 21;7(11):e48478. doi: 10.1371/journal.pone.0048478 (PMC3504044; doi:10.1371/journal.pone.0048478)
Supplement: Table S1 — Main results of the multi-parametric evaluation of treated versus healthy and untreated GRMD dogs. The mean (SD) of each evaluated parameter is provided in this table, at 4 time points of the study: from treatment initiation (2 months) to treatment end (9 months), with two intermediary points (4 and 6 months). The results are given for the treated dogs (GRMDCsA+P) and for the untreated GRMD dogs (GRMDctrl), as well as for the healthy population when this last information is available. (DOCX) [file pone.0048478.s001.docx]

**TABLE S1: Main results of the multi-parametric evaluation of treated versus healthy and untreated GRMD dogs**

|  | | 2 months  Mean (SD) | 4 months  Mean (SD) | 6 months  Mean (SD) | 9 months  Mean (SD) |
| --- | --- | --- | --- | --- | --- |
| Serum CK (UI/L) | GRMD*^ctrl^* | 21484 (6174) | 12662 (5880) | 27327 (20605) | 24136 (14414) |
|  | GRMD*^CsA+P^* | 12142 (4367) | 7138 (2391) | 1446 (1267) | 678 (448) |
| Pathological index (%) | GRMD*^ctrl^* |  |  | 39.2 (19.2) | 33.7 (5.7) |
|  | GRMD*^CsA+P^* |  |  | 36.5 (6.2) | 33.7 (5.9) |
| Fibrosis (%) | GRMD*^ctrl^* |  |  | 29.0 (13.2) | 26.9 (2.8) |
|  | GRMD*^CsA+P^* |  |  | 30.6 (5.6) | 34.9 (7.9) |
| Calcium overloaded fibers (%) | GRMD*^ctrl^* |  |  | 4.5.10^-6^ (7.0.10^-6^) | 0.4.10^-6^ (0.2.10^-6^) |
|  | GRMD*^CsA+P^* |  |  | 2.9.10^-6^ (1.7.10^-6^) | 1.7.10^-6^ (1.4.10^-6^) |
| Calcified fibers (%) | GRMD*^ctrl^* |  |  | 0.1.10^-6^ (0.2.10^-6^) | 0.5.10^-6^ (0.4.10^-6^) |
|  | GRMD*^CsA+P^* |  |  | 2.5.10^-6^ (1.6.10^-6^) | 1.5.10^-6^ (1.0.10^-6^) |
| Mean fiber diameter (µm) | GRMD*^ctrl^* |  |  | 43.8 (4.8) | 44.9 (8.6) |
|  | GRMD*^CsA+P^* |  |  | 34.8 (4.6) | 34.5 (4.3) |
| Type I fibers (%) | GRMD*^ctrl^* |  |  | 60.4 (11.0) | 57.6 (17.2) |
|  | GRMD*^CsA+P^* |  |  | 37.2 (14.9) | 30.6 (12.7) |
| CD4+ cells (/mm²) | GRMD*^ctrl^* |  |  | 3.80 (3.69) | 3.33 (3.03) |
|  | GRMD*^CsA+P^* |  |  | 3.24 (2.46) | 1.88 (2.39) |
| CD8+ cells (/mm²) | GRMD*^ctrl^* |  |  | 0.59 (0.76) | 1.81 (1.81) |
|  | GRMD*^CsA+P^* |  |  | 1.90 (2.48) | 0.90 (0.90) |
| CD11b+ cells (/mm²) | GRMD*^ctrl^* |  |  | 55.9 (19.2) | 78.0 (58.4) |
|  | GRMD*^CsA+P^* |  |  | 104.9 (95.3) | 32.9 (10.1) |
| Relative force (N/kg) | Healthy |  | 0.56 (0.17) | 0.59 (0.08) |  |
|  | GRMD*^ctrl^* |  | 0.33 (0.14) | 0.37 (0.09) | 0.22 (0.19) |
|  | GRMD*^CsA+P^* |  | 0.24 (0.12) | 0.17 (0.06) | 0.15 (0.04) |
| Fatigue index (%) | Healthy |  | 14.2 (21.8) | 15.7 (7.7) |  |
|  | GRMD*^ctrl^* |  | 39.4 (17.5) | 22.3 (19.8) | -2.9 (3.3) |
|  | GRMD*^CsA+P^* |  | 40.1 (13.1) | 50.0 (19.4) | 30.3 (13.8) |
| 100 ms relaxation level (% tetanic force) | Healthy |  | 49.4 (2.0) | 48.5 (4.2) |  |
|  | GRMD*^ctrl^* |  | 42.5 (7.6) | 44.5 (8.3) |  |
|  | GRMD*^CsA+P^* |  | 61.6 (2.3) | 61.7 (1.1) |  |
| Post-tetanic residual contraction (% tetanic force) | Healthy |  | -1.04 (1.02) | 0.06 (0.37) |  |
|  | GRMD*^ctrl^* |  | 5.58 (7.40) | 12.91 (15.41) |  |
|  | GRMD*^CsA+P^* |  | -2.78 (2.24) | -3.6 (0.41) |  |
| Clinical motor score (%) | GRMD*^ctrl^* | 20.7 (5.0) | 44.7 (16.6) | 63.8 (20.0) | 48.6 (9.7) |
|  | GRMD*^CsA+P^* | 23.1 (2.2) | 40.1 (20.8) | 32.6 (15.7) | 36.9 (12.6) |
| Speed/height (/s) | Healthy | 5.68 (0.71) | 4.63 (0.64) | 4.36 (0.26) | 4.39 (0.19) |
|  | GRMD*^ctrl^* | 3.36 (1.01) | 2.35 (0.92) | 2.05 (0.71) | 1.01 (0.65) |
|  | GRMD*^CsA+P^* | 2.74 (0.22) | 5.62 (0.11) | 2.87 (0.27) | 3.00 (0.34) |
| Stride frequency (/s) | Healthy | 2.89 (0.14) | 2.31 (0.10) | 2.12 (0.11) | 2.21 (0.10) |
|  | GRMD*^ctrl^* | 2.49 (0.51) | 2.01 (0.50) | 1.96 (0.49) | 1.74 (0.37) |
|  | GRMD*^CsA+P^* | 2.24 (0.24) | 2.77 (0.57) | 2.13 (0.18) | 2.07 (0.14) |
| Stride length/height | Healthy | 1.97 (0.21) | 1.93 (0.15) | 2.05 (0.08) | 1.99 (0.05) |
|  | GRMD*^ctrl^* | 1.30 (0.22) | 1.14 (0.19) | 1.03 (0.14) | 0.94 (0.26) |
|  | GRMD*^CsA+P^* | 1.18 (0.13) | 1.82 (0.07) | 1.30 (0.07) | 1.45 (0.06) |
| Regularity | Healthy | 197.0 (40.8) | 255.9 (13.4) | 280.1 (37.0) | 269 (34.7) |
|  | GRMD*^ctrl^* | 204.3 (55.3) | 189.7 (44.6) | 195.1 (57.8) | 228.4 (32.6) |
|  | GRMD*^CsA+P^* | 214.9 (41.0) | 189.0 (49.9) | 250.3 (54.5) | 225.2 (77.8) |
| Dorso-ventral/total power (%) | Healthy | 40.3 (6.1) | 49.4 (2.4) | 54.9 (2.3) | 43.3 (3.0) |
|  | GRMD*^ctrl^* | 38.2 (11.4) | 31.8 (13.6) | 34.5 (10.1) | 30.6 (6.4) |
|  | GRMD*^CsA+P^* | 40.6 (8.8) | 28.4 (18.8) | 49.6 (7.8) | 47.3 (4.6) |
| Cranio-caudal/total power (%) | Healthy | 44.0 (6.2) | 39.0 (2.1) | 36.2 (4.3) | 47.8 (3.4) |
|  | GRMD*^ctrl^* | 48.1 (14.6) | 47.1 (15.0) | 46.5 (12.8) | 46.2 (13.0) |
|  | GRMD*^CsA+P^* | 46.8 (14.1) | 51.8 (8.5) | 41.2 (7.5) | 42.3 (6.3) |
| Medio-lateral/total power (%) | Healthy | 15.8 (3.9) | 11.6 (1.4) | 8.9 (3.4) | 8.9 (2.6) |
|  | GRMD*^ctrl^* | 13.7 (7.3) | 21.1 (13.6) | 18.8 (8.1) | 21.7 (8.8) |
|  | GRMD*^CsA+P^* | 12.7 (5.4) | 19.8 (10.6) | 9.1 (1.2) | 10.4 (4.4) |
| Total power (W/kg) | Healthy | 81.5 (13.5) | 96.4 (11.1) | 90.8 (12.5) | 111.4 (12.1) |
|  | GRMD*^ctrl^* | 38.1 (18.3) | 21.6 (11.8) | 24.8 (15.6) | 21.2 (14.6) |
|  | GRMD*^CsA+P^* | 35.3 (3.7) | 76.8 (41.9) | 46.6 (7.8) | 37.5 (5.6) |
| Force index (N/kg) | Healthy | 53.2 (4.8) | 36.7 (4.5) | 49.4 (4.3) | 41.7 (2.0) |
|  | GRMD*^ctrl^* | 38.3 (14.9) | 21.3 (7.3) | 21.9 (8.4) | 22.9 (8.7) |
|  | GRMD*^CsA+P^* | 42.5 (0.4) | 40.9 (10.3) | 41.2 (6.9) | 30.8 (3.3) |
| Gait quality index | Healthy | 1.32 (0.59) | 0.59 (0.18) | 0.78 (0.19) | 0.83 (0.24) |
|  | GRMD*^ctrl^* | 2.98 (0.78) | 3.69 (0.99) | 3.62 (0.72) | 3.74 (0.87) |
|  | GRMD*^CsA+P^* | 3.02 (0.31) | 3.81 (0.79) | 2.09 (0.20) | 2.35 (0.12) |

The mean (SD) of each evaluated parameter is provided in this table, at 4 time points of the study: from treatment initiation (2 months) to treatment end (9 months), with two intermediary points (4 and 6 months). The results are given for the treated dogs (GRMD^CsA+P^) and for the untreated GRMD dogs (GRMD^ctrl^), as well as for the healthy population when this last information is available.
