# Supplementary figures and images for: Effects of an Immunosuppressive Treatment in the GRMD Dog Model of Duchenne Muscular Dystrophy
Source: PLoS One. 2012 Nov 21;7(11):e48478. doi: 10.1371/journal.pone.0048478 (PMC3504044; doi:10.1371/journal.pone.0048478)

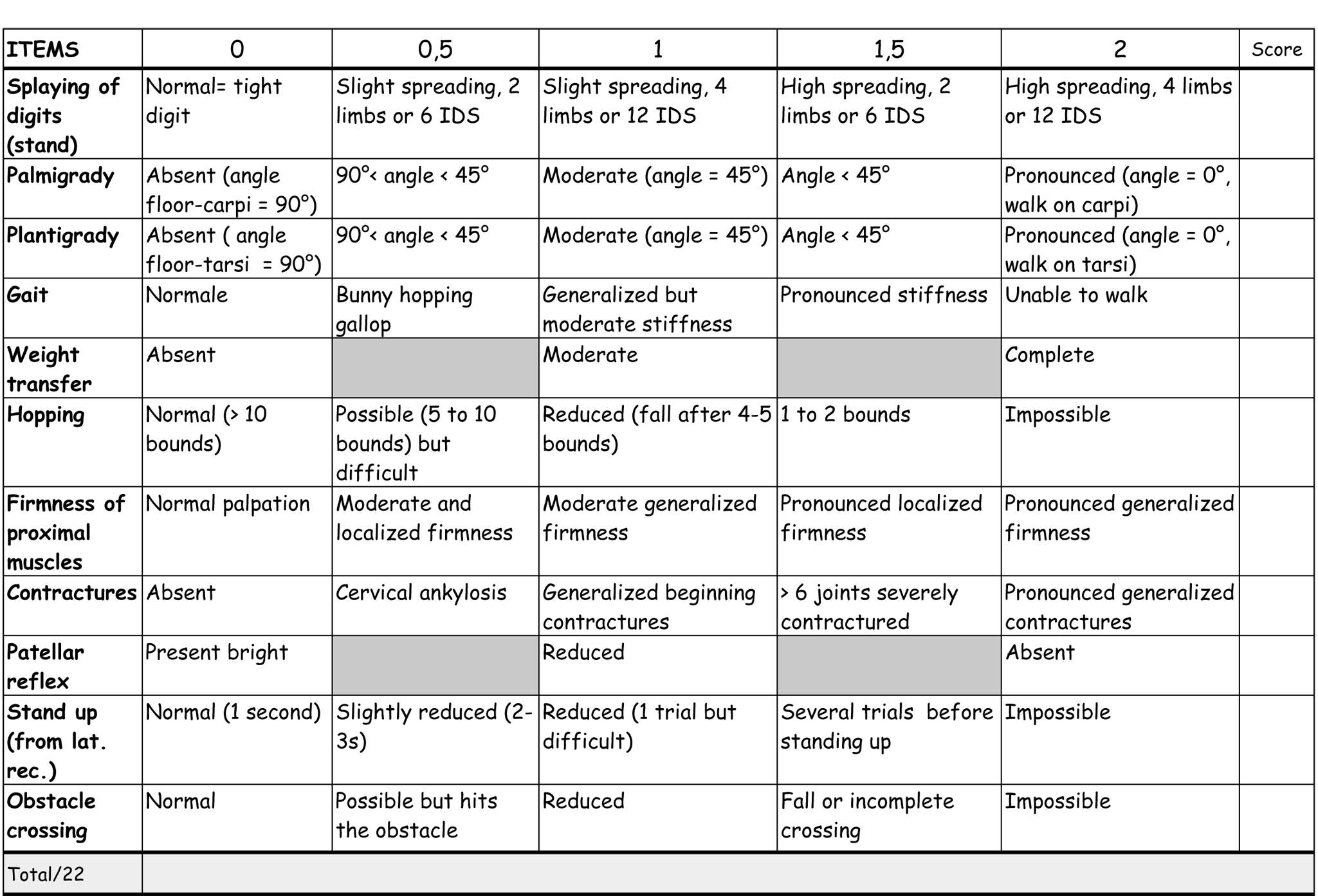

Supplement: Figure S1 — Clinical motor scoring grid. The clinical motor scoring grid encompasses 11 items, allowing the operator to evaluate the dog by observing postural abnormalities, contractures, ambulation, or by performing simple tests as the hopping test or the ability to stand up or cross an obstacle. Each item can be scored from 0 (normal situation) to 2 (the worst situation), giving a score on 22 points, the score 0/22 being the one a healthy dog should obtain, and 22/22 describing the worst motor clinical situation a GRMD could be in. Abbreviations: IDS: inter-digital space; from lat. rec.: from lateral recumbency. (TIF) [file pone.0048478.s002.tif]

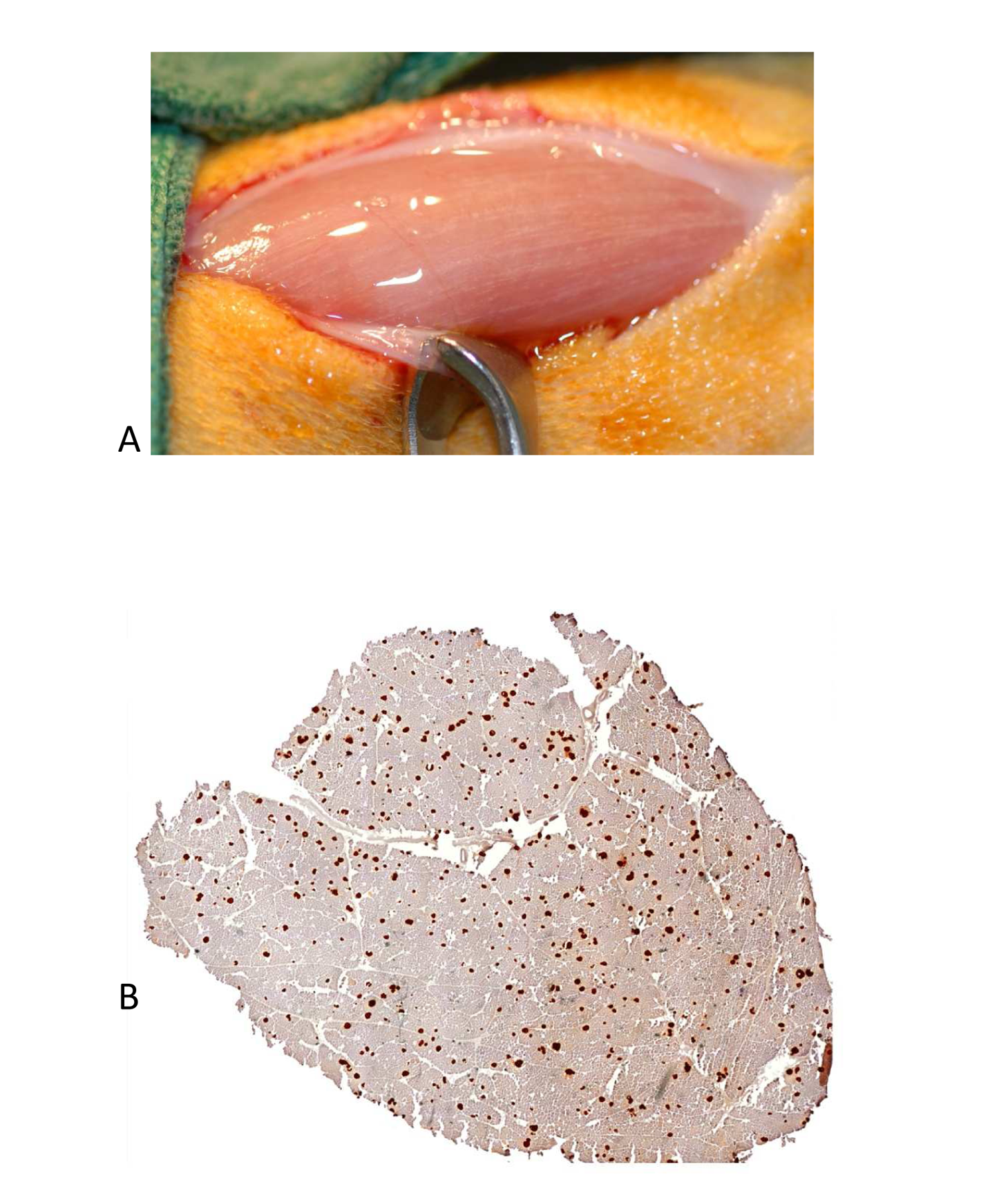

Supplement: Figure S2 — Illustration of the high prevalence of calcified myofibers in treated dogs. A: Macroscopically visible calcified myofibers in a GRMDCsA+P tibialis cranialis muscle. B: Alizarin red S (ARS) staining of a GRMDCsA+P tibialis cranialis muscle biopsy at 6 months, picture of a whole section. The amount of ARS positive fibers is remarkable. (TIF) [file pone.0048478.s003.tif]

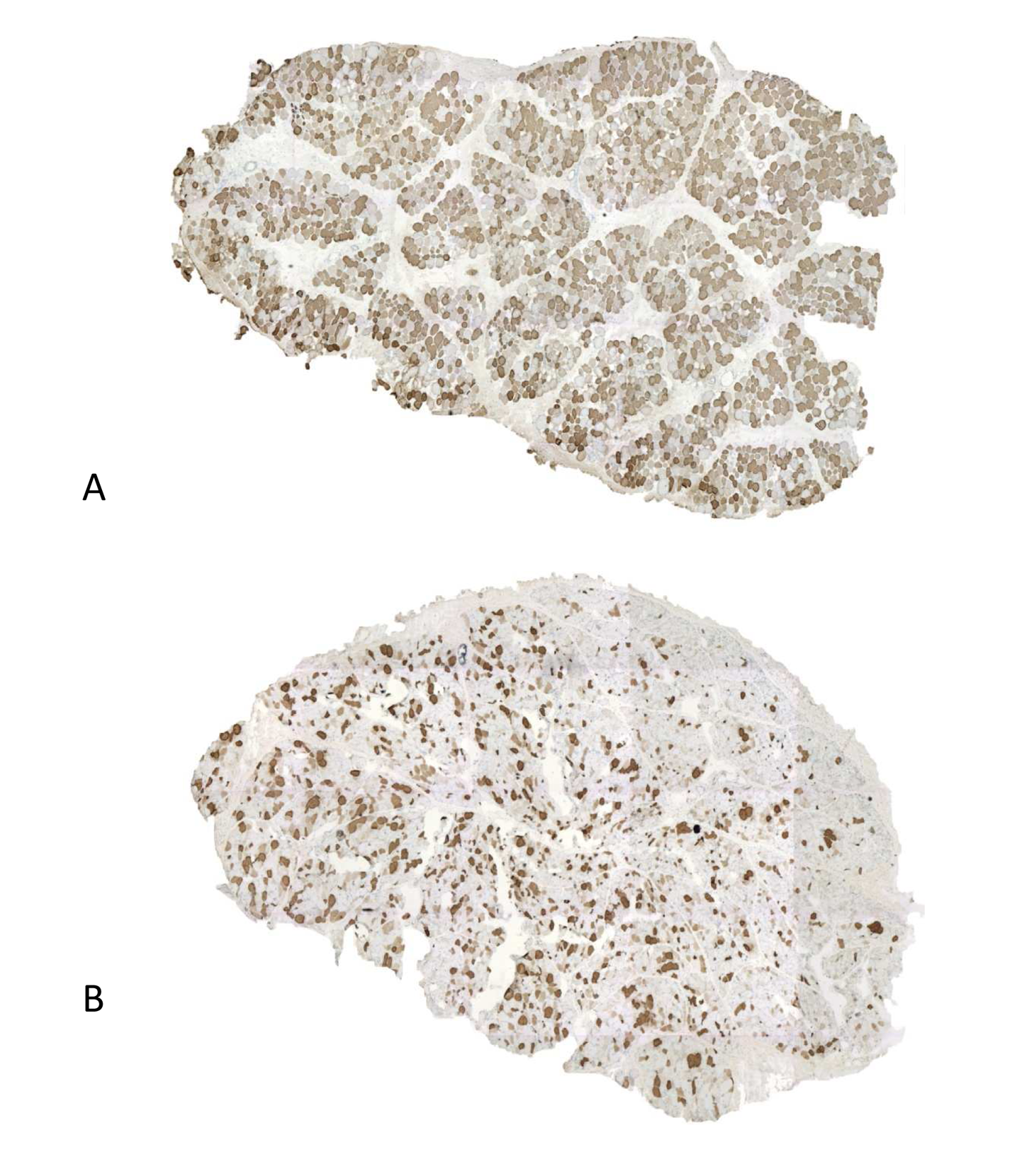

Supplement: Figure S3 — Illustration of the MHCs+ positive fibers rarefaction treated dogs. MHCs immunostaining of a whole GRMDctrl tibialis cranialis biopsy section (A) in comparison with the same immunostaining performed on a whole GRMDCsA+P tibialis cranialis biopsy section at 9 months (B). The loss of MHCs+ fibers predominance in treated dogs is evident. (TIF) [file pone.0048478.s004.tif]
